# Transfer of the Experimental Autoimmune Glaucoma Model from Rats to Mice—New Options to Study Glaucoma Disease

**DOI:** 10.3390/ijms20102563

**Published:** 2019-05-24

**Authors:** Sabrina Reinehr, Jacqueline Reinhard, Susanne Wiemann, Karoline Hesse, Christina Voss, Marcel Gandej, H. Burkhard Dick, Andreas Faissner, Stephanie C. Joachim

**Affiliations:** 1 Experimental Eye Research Institute, University Eye Hospital, Ruhr-University Bochum, In der Schornau 23-25, 44892 Bochum, Germany; sabrina.reinehr@rub.de (S.R.); karoline-hesse@gmx.de (K.H.); christinavoss@gmx.net (C.V.); marcel.gandej@gmx.de (M.G.); burkhard.dick@kk-bochum.de (H.B.D.); 2 Department of Cell Morphology and Molecular Neurobiology, Faculty of Biology and Biotechnology, Ruhr-University Bochum, Universitaetsstrasse 150, 44780 Bochum, Germany; jacqueline.reinhard@rub.de (J.R.); susanne.wiemann@rub.de (S.W.); andreas.faissner@rub.de (A.F.)

**Keywords:** autoimmune glaucoma model, immunization, retinal ganglion cell loss, synapse

## Abstract

Studies have suggested an involvement of the immune system in glaucoma. Hence, a rat experimental autoimmune glaucoma model (EAG) was developed to investigate the role of the immune response. Here, we transferred this model into mice. Either 0.8 mg/mL of the optic nerve antigen homogenate (ONA; ONA 0.8) or 1.0 mg/mL ONA (ONA 1.0) were injected in 129/Sv mice. Controls received sodium chloride. Before and 6 weeks after immunization, the intraocular pressure (IOP) was measured. At 6 weeks, retinal neurons, glia cells, and synapses were analyzed via immunohistology and quantitative real-time PCR (RT-qPCR). Additionally, optic nerves were examined. The IOP stayed in the normal physiological range throughout the study (*p* > 0.05). A significant reduction of retinal ganglion cells (RGCs) was noted in both immunized groups (*p* < 0.001). Remodeling of glutamatergic and GABAergic synapses was seen in ONA 1.0 retinas. Furthermore, both ONA groups revealed optic nerve degeneration and macrogliosis (all: *p* < 0.001). An increase of activated microglia was noted in ONA retinas and optic nerves (*p* < 0.05). Both ONA concentrations led to RGC loss and optic nerve degeneration. Therefore, the EAG model was successfully transferred from rats to mice. In further studies, transgenic knockout mice can be used to investigate the pathomechanisms of glaucoma more precisely.

## 1. Introduction

Glaucoma is a progressive neuropathy with changes in the optic nerve head, gradual retinal ganglion cell (RGC) death, and visual field loss [1]. Although an elevated intraocular pressure (IOP) is the main risk factor, IOP-unrelated pathomechanisms also occur. Since this disease is multifactorial, appropriate models mimicking possible pathological pathways are needed. In the past few years, a rat experimental autoimmune glaucoma model (EAG) was used to identify mechanisms related to immunological alterations in IOP-independent glaucoma. Here, rats were immunized with ocular antigens, such as heat shock proteins (HSPs) or an optic nerve antigen homogenate (ONA). This led to a loss of RGCs and optic nerve degeneration [2,3,4]. Additionally, an enhanced activation of glia cells and complement system proteins could be observed [4,5,6,7,8]. Furthermore, a remodeling of extracellular matrix proteins was noted [9]. So far, the EAG model has only been established in rats. Therefore, we aimed to transfer this model into mice in the study presented here. 

The purpose of this study was to determine if an immunization with ONA leads to RGC death and optic nerve degeneration in mice. Furthermore, other retinal cell types, such as bipolar cells, photoreceptors, and glia cells were analyzed in order to investigate the effects of immunization on these cells. Since a disruption of axonal transport in RGCs seems to be play a role in glaucoma, we aimed to examine different types of synapses in this new model.

Hence, we immunized 129/Sv mice with two different ONA concentrations followed by histological and quantitative real-time PCR (RT-qPCR) analyses after 6 weeks. We could observe a loss of RGCs in combination with optic nerve degeneration in ONA-immunized mice. This was accompanied by a remodeling of synapses in the retina. These results lead to the conclusion that we successfully transferred the EAG model from rats to mice. 

## 2. Results

### 2.1. Intraocular Pressure in Normal Range

Before and 6 weeks after immunization, IOP was measured in all groups (supplement Figure S1A). At baseline, the ONA 0.8 (12.09 ± 0.79 mmHg, *p* = 0.7) as well as the ONA 1.0 group (12.22 ± 0.47 mmHg, *p* = 0.8) revealed no IOP changes compared to controls (13.43 ± 0.23 mmHg). Furthermore, 6 weeks after immunization, the IOP was not altered in both ONA-immunized groups (ONA 0.8: 13.90 ± 0.38 mmHg, *p* = 0.053; ONA 1.0: 13.31 ± 0.60 mmHg, *p* = 0.18) in comparison to the control animals (11.02 ± 0.52 mmHg). 

Retinal cross-sections were stained with hematoxylin and eosin (H&E) and cresyl violet to get an overview of possible changes in structure (supplement Figure S1B). Retinal layers were well defined, and no infiltrates or signs of inflammation were noted. Control retinas and those of both ONA-immunized groups were comparable in regard to layer thickness and structure. 

### 2.2. Loss of Retinal Ganglion Cells

**Figure 1:**
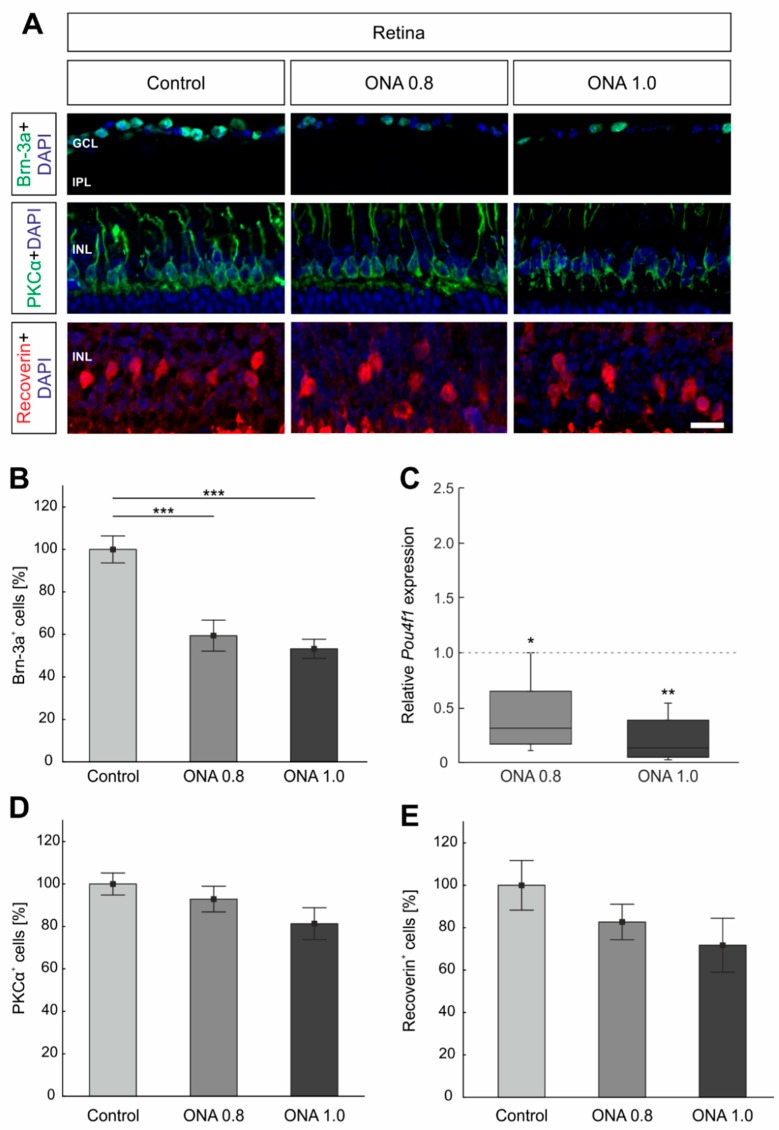
Loss of retinal ganglion cells. (**A**) Retinal cross-sections were stained with antibodies against anti-Brn-3a (retinal ganglion cells—RGCs; green), anti-PKCα (rod bipolar cells; green), and anti-recoverin (cone bipolar cell; red). Cell nuclei were labeled with DAPI (blue). (**B**) The number of Brn-3a^+^ cells was significantly reduced in optic nerve antigen homogenate (ONA) 0.8 and ONA 1.0 animals (both: *p* < 0.001) compared to the control group. (**C**) Additionally, the mRNA expression of *Pou4f1* showed a significant downregulation in ONA 0.8 (*p* = 0.02) and ONA 1.0 retinas (*p* = 0.008). (**D**) Regarding PKCα^+^ cells, no changes could be noted in both immunized groups compared to controls (*p* > 0.05). (**E**) Also, the number of recoverin^+^ cells remained unaltered (*p* > 0.05). Abbreviations: GCL = ganglion cell layer, IPL = inner plexiform layer, INL = inner nuclear layer. The dotted line in C represents the relative expression level of the control group. Values are mean ± SEM for immunohistology and median ± quartile + maximum/minimum for RT-qPCR. Scale bar: 20 µm. * *p* < 0.05, ** *p* < 0.01, *** *p* < 0.001.

To evaluate the number of RGCs, retinal cross-sections were stained with an antibody against Brn-3a after 6 weeks (Figure 1A). Significantly fewer Brn-3a^+^ cells were noted in ONA 0.8 (59.36 ± 7.30%; *p* < 0.001) and ONA 1.0 retinas (53.16 ± 4.50%; *p* < 0.001) compared to controls (100.00 ± 6.37%; Figure 1B). Additionally, RT-qPCR analyses revealed a downregulation of *Pou4f1* mRNA levels in ONA 0.8 (0.31-fold expression; *p* = 0.017) and ONA 1.0 animals (0.14-fold expression; *p* = 0.008; Figure 1C).

### 2.3. No Alterations in Bipolar Cells

To evaluate the number of bipolar cells, retinas were labeled with anti-PKCα (rod bipolar cells) and anti-recoverin (cone bipolar cells; Figure 1A). Staining with PKCα revealed no changes in ONA 0.8 (92.88 ± 6.06%; *p* = 0.7) and ONA 1.0 animals (81.28 ± 7.51%; *p* = 0.1) compared to controls (100.00 ± 5.17%; Figure 1D). Regarding recoverin, both immunized groups (ONA 0.8: 82.69 ± 8.37%, *p* = 0.5; ONA 1.0: 71.72 ± 12.71%, *p* = 0.2) showed no differences in comparison to control retinas (100.00 ± 11.72%; Figure 1E).

### 2.4. Photoreceptors Are Not Affected

**Figure 2:**
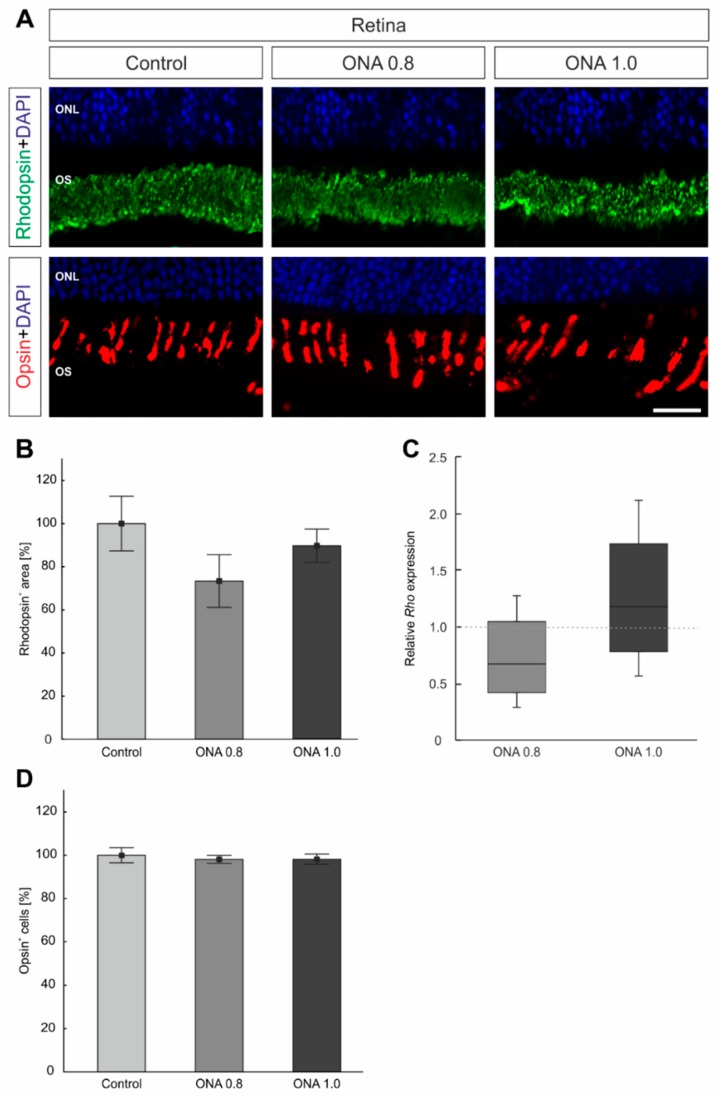
No photoreceptor degeneration. (**A**) Retinas were stained with antibodies against anti-rhodopsin (rods; green) and anti-opsin (cones; red). Cell nuclei were marked in blue. (**B**) Comparable rhodopsin^+^ areas were observed in all groups (*p* > 0.05). (**C**) The RT-qPCR analyses revealed a similar *Rho* mRNA expression in both immunized groups compared to controls (*p* > 0.05). (**D**) The number of opsin^+^ cells was not altered in ONA-immunized animals compared to control (*p* > 0.05). Abbreviations: ONL = inner nuclear layer, OS = outer segment. The dotted line in C represents the relative expression level of the control group. Values are mean ± SEM for immunohistology and median ± quartile + maximum/minimum for RT-qPCR. Scale bar: 20 µm.

L-cones were labeled with anti-opsin and rods were visualized with anti-rhodopsin to analyze whether the immunization affected photoreceptors (Figure 2A). The rhodopsin^+^ area in ONA 0.8 (73.35 ± 12.23%; *p* = 0.2), ONA 1.0 (89.29 ± 7.72%; *p* = 0.7), and control animals (100.00 ± 12.70%; Figure 2B) was comparable. Furthermore, the mRNA level of *Rho* remained unchanged in ONA 0.8 (0.67-fold expression; *p* = 0.1) as well as ONA 1.0 retinas (1.18-fold expression; *p* = 0.4; Figure 2C). Furthermore, similar numbers of opsin^+^ cells were counted in ONA 0.8 (98.11 ± 1.86%; *p* = 0.8), ONA 1.0 retinas (98.17 ± 2.36%, both *p* = 0.8), and controls (100.00 ± 3.47%; Figure 2D). 

### 2.5. Remodeling of Synapses

**Figure 3:**
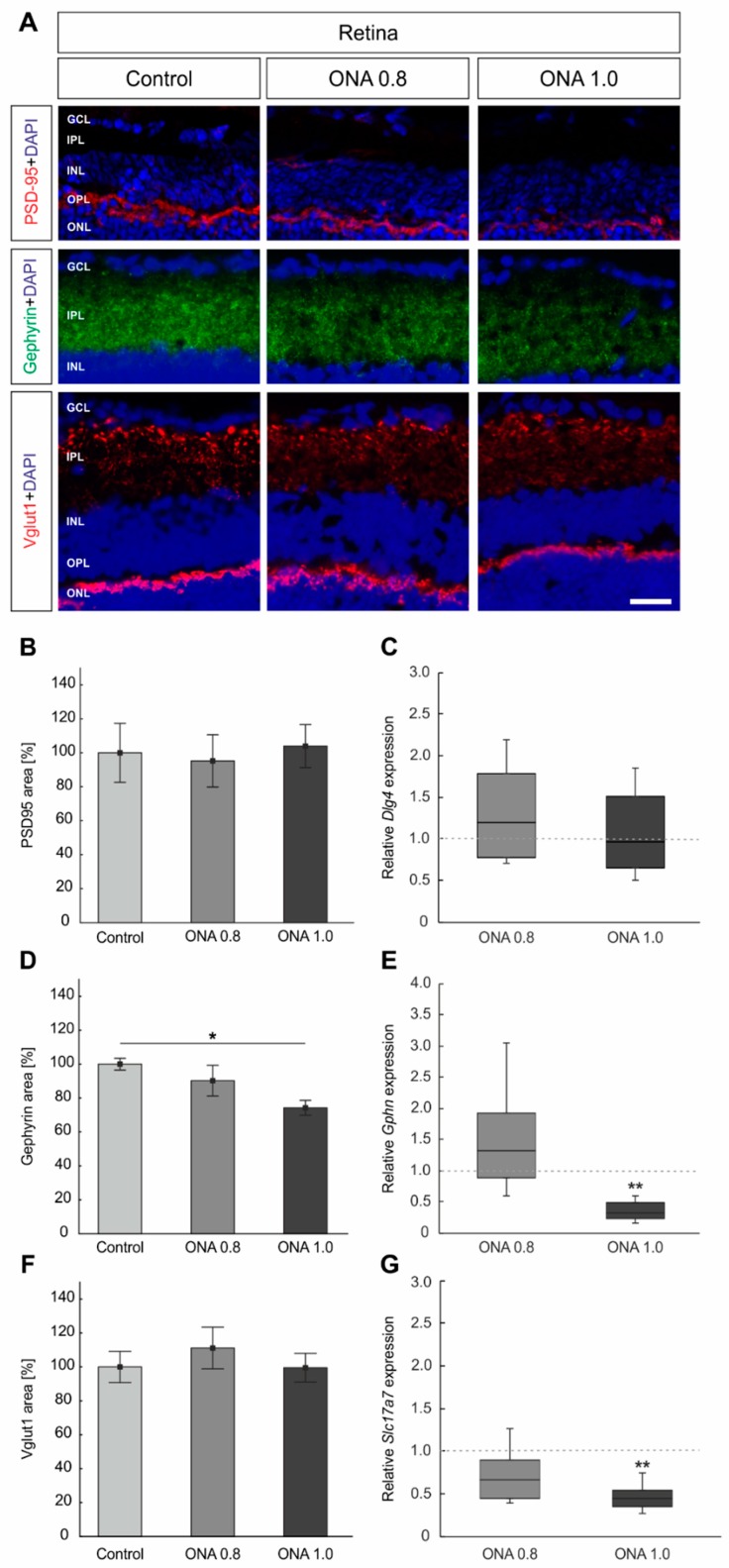
Remodeling of synapses. (**A**) Cross-sections of the retina were stained with antibodies against anti-PSD95 (postsynapses; red), anti-gephyrin (GABAergic synapses; green), and anti-Vglut1 (glutamatergic synapses; red). (**B**) No alterations were noted in PSD95^+^ area in both immunized groups compared to control animals (*p* > 0.05). (**C**) Furthermore, the mRNA expression levels of *Dlg4* (Psd95) were not altered (*p* > 0.05). (**D**) The gephyrin area showed no changes in ONA 0.8 animals compared to controls (*p* > 0.05). In contrast, a significant decrease of gephyrin signal was observed in ONA 1.0 retinas (*p* = 0.02). (**E**) The RT-qPCR analyses of *Gphn* showed no differences in the ONA 0.8 animals (*p* > 0.05). However, a significant downregulation of *Gphn* mRNA was noted in ONA 1.0 retinas (*p* = 0.008). (**F**) The Vglut1^+^ staining area remained unaltered in both ONA immunized groups (*p* > 0.05). (**G**) The mRNA expression of *Slc17a7* did not changed in the ONA 0.8 group (*p* > 0.05). In ONA 1.0 retinas, a significant downregulation of *Slc17a7* mRNA could be noted (*p* = 0.009). Abbreviations: GCL = ganglion cell layer, IPL = inner plexiform layer, INL = inner nuclear layer, OPL = outer plexiform layer, ONL = outer nuclear layer. The dotted line in C, E, and G represents the relative expression level of the control group. Values are mean ± SEM for immunohistology and median ± quartile + maximum/minimum for RT-qPCR. Scale bar: 20 µm. * *p* < 0.05, ** *p* <0.01.

Postsynapses in the retina were labeled with anti-PSD95, GABAergic synapses with anti-gephyrin, and glutamatergic synapses with an antibody against Vglut1 (Figure 3A,B,D,F). Furthermore, the mRNA levels of *Dlg4* (Psd95), *Gphn*, and *Slc17a7* (Vglut1) were analyzed via RT-qPCR (Figure 3C,E,G).

Regarding PSD95, we noted no alterations in the staining area in ONA 0.8 animals (95.23 ± 15.38%) compared to controls (100.00 ± 17.42%; *p* = 0.9; Figure 3B). Furthermore, the PSD95^+^ area in ONA 1.0 retinas (103.98 ± 12.65%; *p* = 0.9) was not modified. In accordance, the RT-qPCR analyses revealed comparable *Dlg4* mRNA levels in both immunized groups (ONA 0.8: 1.2-fold expression; *p* = 0.4; ONA 1.0: 0.96-fold expression; *p* = 0.8; Figure 3C). 

In regard to gephyrin, no changes were observed in the staining area in ONA 0.8 animals (90.20 ± 9.07%; *p* = 0.5) compared to controls (100.00 ± 3.47%; Figure 3D). However, a significantly smaller gephyrin signal area was observed in ONA 1.0 retinas (74.20 ± 4.3%; *p* = 0.02). In the ONA 0.8 groups, the mRNA level of *Gphn* remained unaltered (0.8-fold expression; *p* = 0.3; Figure 3E). In accordance with the histological results, a significant downregulation of *Gphn* mRNA was noted in ONA 1.0 retinas (0.3-fold expression; *p* = 0.008).

The staining area of Vglut1 was comparable in ONA 0.8 retinas (111.15 ± 12.31%; *p* = 0.7) in comparison to controls (100.00 ± 9.16%; Figure 3F). Furthermore, no changes occurred in ONA 1.0 animals (99.52 ± 8.50%; *p* = 0.9). The RT-qPCR analyses of *Slc17a7* also revealed no differences in the ONA 0.8 animals (0.67-fold expression; *p* = 0.1). On the other hand, a significant downregulation of *Slc17a7* mRNA levels was observed in the ONA 1.0 group (0.44-fold; *p* = 0.009; Figure 3G).

### 2.6. Activated Microglia in the Retina

**Figure 4:**
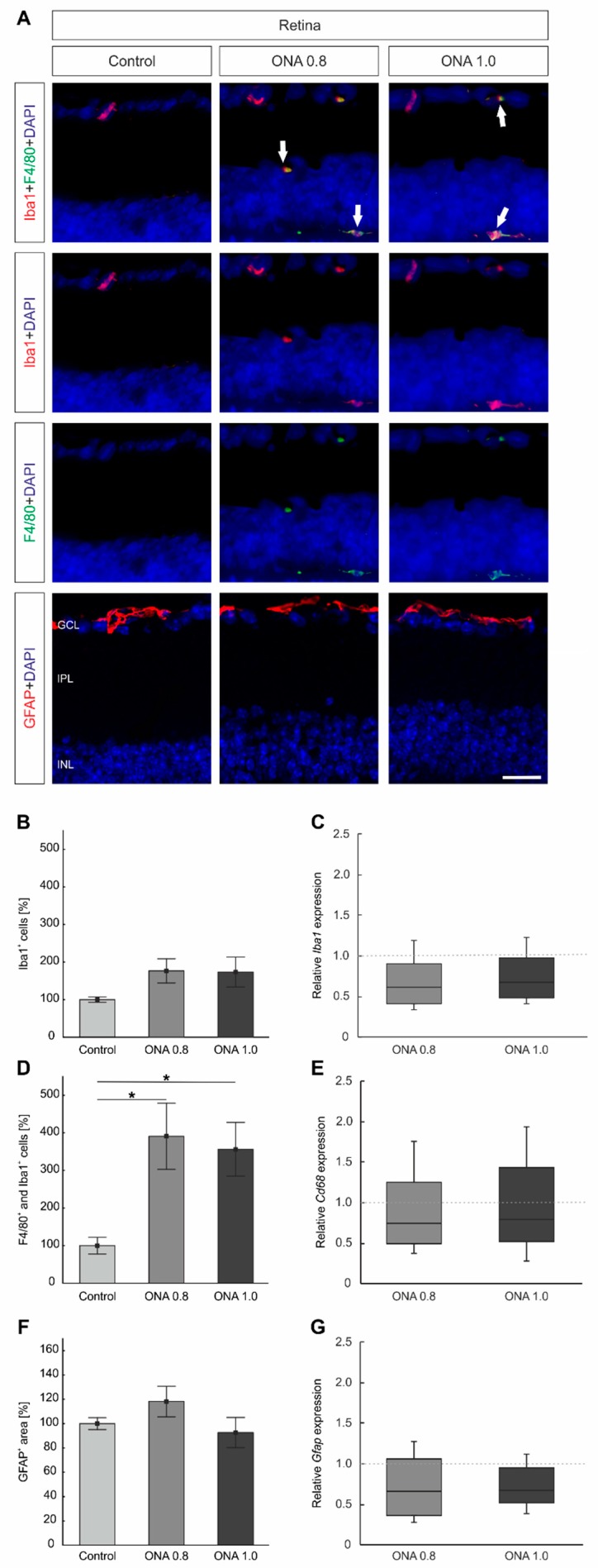
Activated microglia in the retina. (**A**) Retinal cross-sections were stained with antibodies against anti-Iba 1 (all microglia; red) and in combination with anti-F4/80 (active microglia; green). Astrocytes were marked with anti-GFAP (red). DAPI labeled cell nuclei (blue). (**B**) The number of Iba1^+^ cells remained unchanged in both immunization groups compared to control (*p* > 0.05). **(C**) RT-qPCR analyses revealed no differences in *Iba1* expression levels in both ONA-immunized groups (*p* > 0.05). (**D**) Significantly more activated microglia cells could be noted in ONA 0.8 (*p* = 0.02) and ONA 1.0 retinas (*p* = 0.04) compared to controls. (**E**) However, no changes were observed in the *Cd68* mRNA expression in both ONA treated groups (*p* > 0.05). (**F**) The GFAP staining showed no alterations in ONA 0.8 and ONA 1.0 retinas compared to controls (*p* > 0.05). (**G**) The mRNA expression levels of *Gfap* remained unaltered in both immunized ONA groups compared to controls (*p* > 0.05). Abbreviations: GCL = ganglion cell layer, IPL = inner plexiform layer, INL = inner nuclear layer. The dotted line in C, E, and G represents the relative expression level of the control group. Values are mean ± SEM for immunohistology and median ± quartile + maximum/minimum for RT-qPCR. Scale bar: 20 µm. * *p* < 0.05.

In the retina, the whole population of microglia was labeled with anti-Iba1 and activated microglia were visualized by combining this marker with anti-F4/80. Astrocytes were labeled with anti-GFAP (Figure 4A). Additionally, RT-qPCR analyses were performed for *Iba1*, *Cd68*, and *Gfap* (Figure 4C,E,G). 

The number of Iba1^+^ cells remained unchanged in ONA 0.8 retinas (176.42 ± 32.14%) compared to controls (100.00 ± 7.30%; *p* = 0.2). Furthermore, no differences were noted in ONA 1.0 animals (173.37 ± 39.65%; *p* = 0.2; Figure 4B). In agreement with these findings, the mRNA expression levels of *Iba1* was neither altered in ONA 0.8 (0.6-fold expression; *p* = 0.2) nor in ONA 1.0 retinas (0.7-fold expression; *p* = 0.3; Figure 4C).

A significantly enhanced number of F4/80^+^ and Iba1^+^ cells could be detected in ONA 0.8 (391.05 ± 87.95%; *p* = 0.02) and ONA 1.0 retinas (356.32 ± 71.46%; *p* = 0.04) compared to controls (100.00 ± 22.36%; Figure 4D). However, the RT-qPCR analyses of *Cd68* mRNA levels revealed no changes in the ONA 0.8 group (0.7-fold expression; *p* = 0.2). No alterations were noted in ONA 1.0 retinas (0.8-fold expression; *p* = 0.4; Figure 4E).

In regard to retinal GFAP staining, no alterations were observed in ONA 0.8 (118.20 ± 12.61%; *p* = 0.4) and ONA 1.0 retinas (92.63 ± 12.40%; *p* = 0.9) compared to controls (100.00 ± 4.91%; Figure 4 F). Furthermore, RT-qPCR analyses revealed no changes in *Gfap* mRNA levels in ONA 0.8 (0.7-fold expression; *p* = 0.1) and ONA 1.0 retinas (0.7-fold expression; *p* = 0.09) compared to controls (Figure 4G). 

### 2.7. Optic Nerve Degeneration

**Figure 5:**
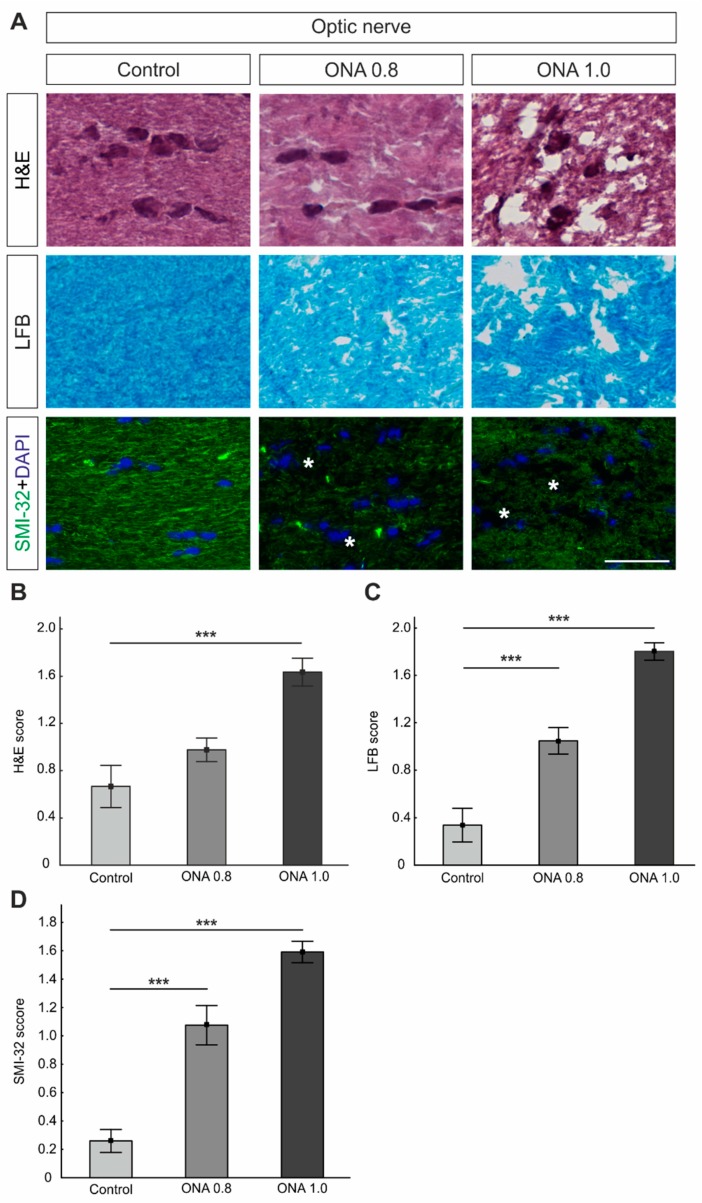
Optic nerve degeneration. (**A**) Optic nerves were stained with hematoxylin and eosin (H&E) and luxol fast blue (LFB). Additionally, neurofilaments were labeled with an antibody against anti-SMI-32 (green). Cell nuclei were marked with DAPI (blue). Asterisks point towards disrupted filaments. (**B**) The ONA 0.8 group and control group had a similar H&E score (*p* > 0.05), while a significantly higher score was noted in ONA 1.0 optic nerves (*p* < 0.001). (**C**) The LFB staining revealed an interruption of myelin sheaths in both ONA immunized groups (both: *p* < 0.001). (**D**) Additionally, we noted a higher SMI-32 score in the ONA 0.8 as well as in the ONA 1.0 optic nerves (*p* < 0.001). Values are mean ± SEM. Scale bar: 20 µm. *** *p* < 0.001.

Longitudinal optic nerve sections were stained with H&E to evaluate the degree of cellular infiltration and with luxol fast blue (LFB) to analyze the extent of demyelination. Additionally, optic nerves were labeled with anti-SMI-32 to detect possible changes in the neurofilament (Figure 5A). 

The evaluation of the H&E staining showed no alterations in the ONA 0.8 optic nerves (mean score: 0.98 ± 0.09; *p* = 0.2). However, significantly more cellular infiltrations were observed in the ONA 1.0 group (1.63 ± 0.12; *p* < 0.001) in comparison to controls (0.67 ± 0.18; Figure 5B). 

Regarding the LFB staining, a significant demyelination was noted in both immunized groups (mean score: ONA 0.8: 1.05 ± 0.11; ONA 1.0: 1.80 ± 0.07; both *p* < 0.001) in comparison to control optic nerves (0.34 ± 0.14; Figure 5C). 

A disruption of SMI-32^+^ neurofilaments was noted in ONA 0.8 optic nerves (mean score: 1.08 ± 0.14; *p* < 0.001). Furthermore, a significantly higher SMI-32 score was observed in the ONA 1.0 animals (1.59 ± 0.08; *p* < 0.001) compared to controls (0.26 ± 0.08; Figure 5D).

### 2.8. Glia Activation in the Optic Nerves

**Figure 6:**
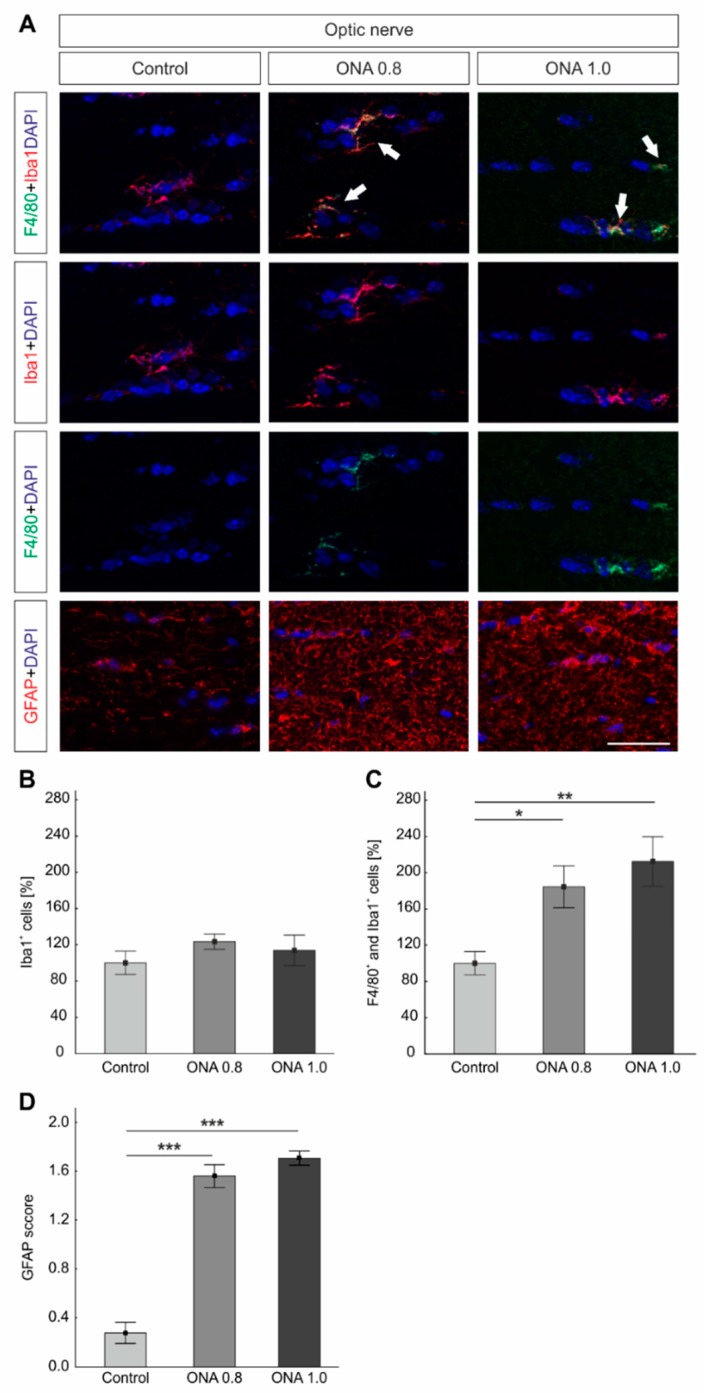
Enhanced glia cells in the optic nerve. (**A**) The total number of microglia was labeled with anti-Iba1 (red) and active microglia in combination with anti-F4/80 (green). Astrocytes were stained with anti-GFAP (red). Cell nuclei were marked in blue. Arrows pointed towards co-labeling of Iba1^+^ and F4/80^+^ cells. (**B**) No changes were noted in the number of Iba1^+^ cells in both immunized groups compared to controls (*p* > 0.05). (**C**) Regarding activated microglia, significantly more F4/80^+^ and Iba1^+^ cells were noted in the ONA 0.8 (*p* = 0.04) and ONA 1.0 optic nerves (*p* = 0.006) compared to controls. (**D**) The GFAP score was significantly higher in the ONA 0.8 as well as in the ONA 1.0 optic nerves (both: *p* < 0.001). Values are mean ± SEM. Scale bar: 20 µm. * *p* < 0.05, ** *p* < 0.01, *** *p* < 0.001.

The whole amount of microglia was labeled with anti-Iba1 and activated microglia were additionally marked with anti-F4/80. Macroglia in the optic nerves were visualized with anti-GFAP (Figure 6A). 

The number of Iba1^+^ microglia was comparable in ONA 0.8 (113.75 ± 16.85%; *p* = 0.7) and ONA 1.0 optic nerves (123.36 ± 8.58 cells/image; *p* = 0.4), when compared to controls (100.00 ± 12.82; Figure 6A,B).

Regarding activated microglia, significantly more F4/80^+^ and Iba1^+^ cells were noted in the ONA 0.8 group (184.47 ± 23.20%; *p* = 0.04) in comparison to controls (100.00 ± 12.90%). Furthermore, a significant increase in activated microglia was observed in ONA 1.0 optic nerves (212.42 ± 27.41%; *p* = 0.006; Figure 6C). 

The GFAP score was significantly higher in the ONA 0.8 (mean score: 1.56 ± 0.93; *p* < 0.001) and in the ONA 1.0 group (1.71 ± 0.06, *p* < 0.001) than in the control group (0.23 ± 0.08; Figure 6D).

## 3. Discussion

### 3.1. Glaucomatous Damage in EAG Mice

The European Glaucoma Society defines glaucoma as a chronic, progressive neuropathy with morphological changes at the optic nerve head and retinal nerve fiber layer, associated with RGC death and visual field loss [1]. We therefore analyzed both tissues, retina and optic nerve, in this study to confirm glaucomatous damage in mice. Six weeks after immunization, a loss of RGCs was noted in both ONA groups. Additionally, optic nerve degeneration was observed. In rats, studies showed that ONA treatment leads to RGC death, starting 22 days after immunization [3,4,6]. To ensure degenerative effects in our mice, investigations were carried out 6 weeks following immunization. We conclude that the transfer of the EAG model from rats to mice was successful. In future, the 129/Sv mouse strain can be used for knockout studies in combination with the autoimmune glaucoma model. This could help to test hypotheses about glaucoma pathologies affected by genetic alterations. 

To investigate the mechanisms occurring during glaucoma more precisely, we additionally analyzed a possible remodeling of synapses as well as glia cell alterations. 

### 3.2. Synaptic Alterations after Immunization

Previously, it was noted that a disruption of the axonal transport in RGCs might represent an early event in glaucoma disease [10]. In the rat EAG model, we could detect a loss of the presynaptic active zone protein bassoon and the postsynaptic protein PSD-95 [11]. These alterations were found 4 weeks after immunizing with S100B in combination with HSP27. In the study presented here, mice immunized with ONA did not display any alterations regarding PSD-95 expression 6 weeks after immunization. Since PSD-95 labels post-synapses of the photoreceptors, it is possible that these are not affected by ONA immunization at this point in time. However, remodeling of GABAergic and glutamatergic synapses was noted. In Morbus Alzheimer, synapse and dendritic spine loss appear in proximity to amyloid beta plaques [12,13]. For example, an increased amyloid pathology in brains with Morbus Alzheimer is correlated with a diminished Vglut1 protein localization, a glutamatergic transporter [14]. A decrease of Vglut1 could also be observed in retinas of an animal model where the pathological effects of apolipoprotein 4 were investigated [15]. A downregulation of *Slc17a7* mRNA levels was noted in the higher concentrated ONA group in our study, assuming that it might also play a crucial role in glaucoma neurodegeneration. 

Gephyrin is a postsynaptic anchor protein, which tethers glycine and GABA_A_ receptors to the cytoskeleton [16], and glycine subtypes are present in the brain and spinal cord [17,18]. Gephyrin immunoreactivity was found in co-localization with amyloid plaques in post-mortem tissue of Morbus Alzheimer patients [17,19]. Besides, gephyrin dysfunctions are also linked to other neurological diseases, like stiff-person syndrome, hyperekplexia, molybdenum cofactor deficiency, schizophrenia, and autism [20,21,22,23]. In the retina, clusters of glycine and GABA_A_ receptors are expressed by RGCs [24]. In our study, we could demonstrate a downregulation of gephyrin in the ONA 1.0 group. Possibly, the loss of RGCs is accompanied with a lower synaptic immunoreactivity of gephyrin.

### 3.3. Reactive Gliosis in the Optic Nerves

It is known that in response to an injury in the central nervous system, astrocytes become reactive [25,26]. In the retina, Müller cells are specialized glia cells contacting retinal neuron somata and processes, providing stability to the neural tissue [27,28]. As in astrocytes, Müller cell proliferation is increased in pathologic eye conditions, such as retinal detachment, ischemia, diabetic retinopathy, or glaucoma [27,29,30,31]. However, until now, it remains unclear whether gliosis is neurodegenerative or neurodestructive [32]. The evaluation of GFAP, expressed by astrocytes and activated Müller cells [29,33], revealed no changes in the retina in our study. In EAG rats on the other hand, 4 weeks after immunization with ONA, enhanced GFAP levels were observed via Western blot in the retina but not in immunohistological analyses [4]. However, a similar number of astrocytes was also noted in a mouse OHT study two weeks after lasering [34]. The authors postulated that in the OHT eyes, a reactive, non-proliferative gliotic response occurred. This was also reported in other mouse and rat studies relating to glaucoma [35,36,37]. A non-proliferative response seems to be the consequence of a slow degeneration, while rapid damages lead to macroglia proliferation [35,37,38]. Furthermore, it is known that astrocytes exhibit multiple phases of remodeling during neurodegeneration [39]. In contrast to the retina, a strong increase of GFAP was noted in the ONA optic nerves in our study. Studies demonstrated that astrocytes in the optic nerve head respond strongly after glaucomatous damage [40]. Furthermore, in humans with a chronic elevated IOP and a moderate or advanced glaucomatous axonal damage, an increased immunoreactivity of GFAP is observable [41,42]. It remains unknown, whether an astrogliosis proceeds to promote detrimental effects on neurons or whether they have a neuroprotective role [43]. It is assumed that in the initial phase of the disease, reactive astroglia have a beneficial role, while with disease progression, these astroglia become neurodestructive [40,44]. The present paradigm could be useful to explore the pharmacological profile of novel anti-glaucoma molecules with a potential protection effect on retina along with an effect on IOP such as sigma receptor ligands [45,46].

Microglia are the resident macrophages of the retina and play an important role in the defense mechanisms of the immune system. Under normal conditions, microglia monitor and remove cellular detritus and maintain cellular homeostasis [47,48,49]. In glaucoma, several studies noted an increase in microglia numbers after induced hypertension or optic nerve damage [50,51,52]. In early stages of the EAG glaucoma rat model, a strong microglia response could be noted at 14 days [4]. Additionally, a decrease of this activation was described between 3 and 12 weeks after optic nerve transection [50]. In the study presented here, we could detect more activated microglia in both retinas and optic nerves. Our results indicate a contribution of the microglia in the EAG model. 

## 4. Methods

### 4.1. Animals

All procedures concerning animals adhered to the ARVO statement for the use of animals in ophthalmic and vision research. All experiments involving animals were approved by the animal care committee of North Rhine-Westphalia, Germany (approval code: 84-02.04.2013.A291; July 2013). 129/Sv (129S2/SvPasCrl) mice were kept under environmentally controlled conditions with free access to chow and water.

### 4.2. Immunization

The preparation and immunization of ONA was carried out as previously described [3,53]. 129/Sv mice received an intraperitoneal injection with either 0.8 mg/mL (ONA 0.8) or 1 mg/mL ONA (ONA 1.0). The antigen was mixed with incomplete Freund’s adjuvant (50 µL; Sigma-Aldrich, St. Louis, MO, USA). The animals of the control group were injected with NaCl in Freund’s adjuvant. Additionally, all mice received 1 µg pertussis toxin (Sigma-Aldrich) intraperitoneally on days 0 and 2 [54].

### 4.3. Measurement of Intraocular Pressure

IOP of both eyes in all animals was measured before and 6 weeks after immunization using a rebound tonometer (TonoLab, Icare, Vantaa, Finland) as described previously (*n* = 3–5/group) [4,7]. For this procedure, mice were anesthetized with a ketamine (Ratiopharm, Ulm, Germany)/xylazine (Bayer healthcare, Berlin, Germany) cocktail (120/16 mg/kg). All measurements were performed by one examiner at the same time of the day. For each analysis, ten measurements per eye were calculated, and the average of the both eyes was used. 

### 4.4. Retina and Optic Nerve Histology

After 6 weeks, retinas and optic nerves were fixed in 4% paraformaldehyde for 1 (retina) or 2 h (optic nerves), dehydrated in sucrose, and embedded in Tissue Tek (Thermo Fisher, Waltham, CA, USA). Cross-sections of the retina (10 µm) and longitudinal optic nerve sections (4 µm) were cut with a Cryostat (Thermo Fisher) and mounted on Superfrost slides (Thermo Fisher). 

### 4.5. Immunohistology 

In order to identify different cell types, specific immunofluorescence antibodies were applied (*n* = 6–7/group; Table 1) [5]. Briefly, retinal cross-sections and longitudinal optic nerve sections were blocked with a solution containing 10–20% donkey and/or goat serum and 0.1% or 0.2% Triton-X in PBS. Primary antibodies were incubated at room temperature overnight. Incubation using corresponding secondary antibodies was performed the next day for 1 h. Nuclear staining with 4’,6 diamidino-2-phenylindole (DAPI, Serva Electrophoresis, Heidelberg, Germany) was included to facilitate the orientation on the slides. Negative controls were performed by using only secondary antibodies. 

### 4.6. Histological Examination 

All photographs were taken with a fluorescence microscope (Axio Imager M1 or M2, Zeiss, Oberkochen, Germany). Two photos of the peripheral and two of the central part of each section were captured. The images were transferred to Corel Paint Shop Pro (V13, Corel Corporation, Ottawa, ON, Canada), and equal excerpts were cut out [11]. Afterwards, RGCs, bipolar cells, microglia, and photoreceptor cells were counted using ImageJ software (National Institute of Health, Bethesda, MD, USA). Data were transferred to Statistica software (V13, Dell, Round Rock, TX, USA) for further analysis. GFAP, synapses, and rhodopsin were evaluated through area analyses using an ImageJ macro [11,55]. Briefly, images were transformed into grayscale. To minimize interference with background labeling, a rolling ball radius was subtracted (Table 2). Then, for each picture, a suitable lower and upper threshold was set. The ideal threshold was obtained when the grayscale picture and the original one corresponded. Afterwards, the mean value of the lower threshold was calculated, and this number was used for the final analysis. The percentage of the labeled area was measured between these defined thresholds (Table 2). Data were transferred to Statistica software for further analysis.

### 4.7. Histopathological Staining and Scoring 

Retinal cross-sections were stained with hematoxylin and eosin (H&E, Merck, Burlington, MA, USA) and cresyl violet (Sigma-Aldrich) to be able to detect any signs of inflammation or changes in retinal structure [56]. To evaluate the extent of cellular infiltration, longitudinal cryo-sections of optic nerves were stained with H&E. The degree of demyelination was examined via LFB (RAL Diagnostics, Martillac Cedex, France) [57]. After staining, ethanol was used for dehydration of the sections, followed by incubation in xylene (Merck) and coating with Eukitt (VWR, Langenfeld, Germany). 

Three images of each optic nerve section (anterior, medial, and posterior) were taken with an Axio Imager M1 microscope at a 400x magnification (six sections per animal).

To examine the extent of inflammatory cell infiltration, an established score was used [58,59]: 0 = no infiltration, 1 = mild cellular infiltration, 2 = moderate infiltration, 3 = severe infiltration, and 4 = massive infiltration with formation of cellular conglomerates. Regarding the degree of demyelination, LFB-stained sections were assessed as previously described [59]: 0 = no demyelination, 0.5 = small holes, 1 = moderate demyelination, 1.5 = bigger holes, and 2 = severe demyelination up to complete loss of structural integrity. Data were transferred to Statistica software for further analysis.

### 4.8. Quantitative Real-Time PCR

Both retinas of each animal (5 animals/group) were pooled for RNA preparation and cDNA synthesis as previously described [5,60]. The designed oligonucleotides for the quantitative real-time-PCR (RT-qPCR) are shown in Table 3. *ß-actin* and *Cyclophilin* (*Ppid*) served as reference genes for retinal analysis. The RT-qPCR was performed using DyNAmo Flash SYBR Green (Thermo Scientific) on the PikoReal RT-qPCR Cycler (Thermo Scientific) [61,62]. Values were transferred to REST© software (Qiagen, Hilden, Germany) for further analysis.

### 4.9. Statistics

Immunohistological data are presented as mean ± SEM. Cell counts and evaluated area fractions were compared by ANOVA followed by Dunnet’s post-hoc. Here, controls were set to 100%. Regarding RT-qPCR, data are presented as median ± quartile + minimum/maximum and were assessed using REST© software [63]. *p*-values below 0.05 were considered statistically significant.

## 5. Conclusions

In the current study, the rat autoimmune glaucoma could successfully be transferred to mice. This offers many more possibilities to investigate the pathomechanisms occurring in glaucoma in future knockout studies. Furthermore, this study provides novel insights into synaptic degeneration in the autoimmune glaucoma model. 

## Figures and Tables

**Table 1:** Primary and secondary antibodies used for immunohistochemistry.

	Primary antibodies				Secondary antibodies				
Antibody	Company	Catalog Number	Tissue	Dilution	Antibody	Company	Catalog Number	Tissue	Dilution
Anti-Brn-3a	Santa Cruz	sc-31984	Retina	1:100	Donkey anti-goat Alexa Fluor 488	Dianova	705-545-147	Retina	1:500
Anti-F4/80	AdB Serotec	MCAA97G	Retina	1:100	Donkey anti-rat Alexa Fluor 488	Thermo Fisher	A-21208	Retina	1:500
			Optic nerve					Optic nerve	
Anti-gephyrin	SySy	147008	Retina	1:500	Donkey anti-rabbit Alexa Fluor 488	Jackson ImmunoResearch	711-547-003	Retina	1:500
Anti-GFAP	Millipore	AB5541	Retina	1:250	Donkey anti-chicken Cy3	Millipore	AP194C	Retina	1:500
			Optic nerve	1:500				Optic nerve	
Anti-Iba1	Wako Chemicals	019-19741	Retina	1:500	Donkey anti-rabbit Alexa Fluor 555	Invitrogen	A31572	Retina	1:500
			Optic nerve					Optic nerve	
Anti-opsin	Millipore	AB5405	Retina	1:500	Donkey anti-rabbit Alexa Fluor 555	Invitrogen	A31752	Retina	1:500
Anti-PKCα	Santa Cruz	sc-8393	Retina	1:500	Goat anti-mouse Alexa Fluor 488	Invitrogen	A11029	Retina	1:500
Anti-PSD95	Calbiochem	CP35	Retina	1:200	Goat anti-mouse Alexa Fluor 555	Invitrogen	A21424	Retina	1:500
Anti-recoverin	Millipore	AB5585	Retina	1:1000	Donkey anti-rabbit Alexa Fluor 555	Invitrogen	A31572	Retina	1:500
Anti-rhodopsin	Abcam	ab3267	Retina	1:400	Goat anti-mouse Alexa Fluor 488	Invitrogen	A11029	Retina	1:500
Anti-SMI-32	Biolegend	801701	Optic nerve	1:2000	Goat anti-mouse Alexa Fluor 488	Invitrogen	A11029	Optic nerve	1:500
Anti-Vglut1	SySy	135316	Retina	1:500	Donkey anti-chicken Cy3	Millipore	AP194C	Retina	1:500

**Table 2:** Adjustments of ImageJ macro for the area analysis. The background subtraction as well as the lower and the upper thresholds are listed.

Protein	Background Subtraction (Pixel)	Lower Threshold	Upper Threshold
Gephyrin	50	7.28	75.41
GFAP	20	8.30	176.00
PSD95	50	5.94	259.67
Rhodopsin	50	9.27	254.87
VGlut1	50	4.80	69.17

**Table 3:** Sequences of oligonucleotides. The listed oligonucleotide pairs were used in quantitative real-time PCR experiments, while *β-actin* and *Cyclophilin (Ppid)* served as housekeeping genes. The predicted amplicon sizes are given. Abbreviations: F = forward, R = reverse, acc. no. = accession number, bp = base pair.

Gene	Forward (F) and Reverse (R) Oligonucleotides	GenBank acc. No.	Amplicon Size
*β-actin*-F *β-actin*-R	ctaaggccaaccgtgaaaag accagaggcatacagggaca	NM_007393.5	104 bp
*Cd68*-F *Cd68*-R	tgatcttgctaggaccgctta taacggcctttttgtgagga	NM_001291058.1	66 bp
*Dlg4-*F *Dlg4*-R	cggatgaagatggcgatag tctgtgcgagaggtagcaga	NM_007864.3	110 bp
*Gphn*-F *Gphn*-R	tgatcttcatgctcagatcca ttgcaaatgttgttggcaag	NM_145965.2	68 bp
*Gfap*-F *Gfap*-R	acagactttctccaacctccag ccttctgacacggatttggt	NM_010277.3	63 bp
*Iba1*-F *Iba1*-R	ggatttgcagggaggaaaa tgggatcatcgaggaattg	D86382.1	92 bp
*Pou4f1*-F *Pou4f1*-R	ctccctgagcacaagtaccc ctggcgaagaggttgctc	AY706205.1	98 bp
*Ppid*-F *Ppid*-R	aaggatggcaaggattgaaa ctttaagcaattctgcctgga	NM_026352	105 bp
*Rho*-F *Rho*-R	tgtggtcttcacctggatcat gaacattgcatgccctcag	NM_145383.1	90 bp
*Slc17a7*-F *Slc17a7*-R	gtgcaatgaccaaggacaag agatgacaccgccgtagtg	NM_182993.2	103 bp

## Supplementary Materials

Supplementary materials can be found at https://www.mdpi.com/1422-0067/20/10/2563/s1.
