# Supplementary material for: Transfer of the Experimental Autoimmune Glaucoma Model from Rats to Mice—New Options to Study Glaucoma Disease
Source: Int J Mol Sci. 2019 May 24;20(10):2563. doi: 10.3390/ijms20102563 (PMC6566658; doi:10.3390/ijms20102563)
Supplement: Supplementary file 1 [file ijms-20-02563-s001.pdf]

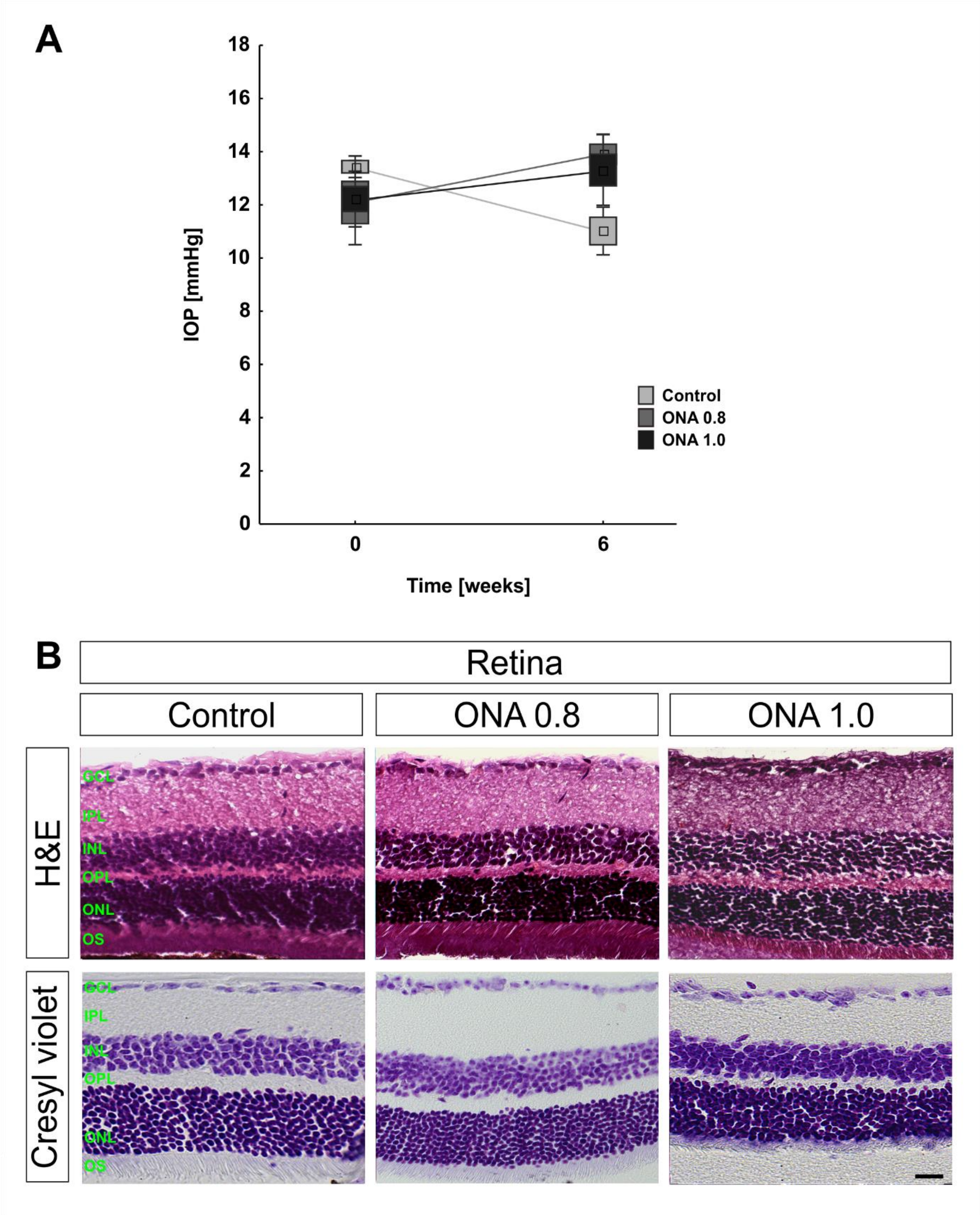

Supplement Figure S1: IOP in normal range. (**A**) IOP was measured before and 6 weeks after immunization. No changes could be observed within all groups at both points in time (p>0.05). (**B**) Retinal cross-sections were stained with H&E and cresyl violet. Retinas of the immunized groups showed no infiltrations or signs of inflammation. Also, the retinal structure was intact and comparable with the control retinas. Abbreviations: GCL=ganglion cell layer, IPL=inner plexiform layer, INL=inner nuclear layer, OPL=outer plexiform layer, ONL=outer nuclear layer, OS=outer segment. Values are mean±SEM±SD. Scale bar: 20 μm.
